# Metformin + Insulin vs. Insulin for GDM and T2DM during pregnancy: systematic review and meta-analysis

**DOI:** 10.61622/rbgo/2026rbgo18

**Published:** 2026-05-12

**Authors:** Christiano Hallack, Bernardo Vieira Nogueira, Maressa Bomfim, Nathália Meireles, Wellington Martins

**Affiliations:** 1 Centro Universitário Serra dos Órgãos Teresópolis RJ Brazil Centro Universitário Serra dos Órgãos, Teresópolis, RJ, Brazil.; 2 SEMEAR Fertilidade Ribeirão Preto SP Brazil SEMEAR Fertilidade, Ribeirão Preto, SP, Brazil.

**Keywords:** Gestational diabetes mellitus, Type 2 diabetes mellitus, Pregnancy complications, Pregnancy outcome, Fetal outcome, Antidiabetic agents, Hypoglycemic agents

## Abstract

**Objective:**

Evaluate the effectiveness and safety of adding metformin to insulin (M+I) versus insulin alone for pregnant women with type 2 diabetes mellitus (T2DM) or gestational diabetes mellitus (GDM), focusing on stillbirth as the primary outcome.

**Sources of data:**

PubMed, Embase, and Cochrane Central were searched. No date limits. Last search: January 2025.

**Eligibility criteria:**

Randomized clinical trials including women with T2DM or GDM were eligible. Trials restricted to type 1 diabetes were excluded.

**Data collection and synthesis:**

Two reviewers extracted maternal and neonatal outcomes and assessed risk of bias with the Cochrane RoB 2 tool. Evidence certainty was graded using GRADE. Data were pooled with random-effects models and reported as risk ratios (RRs) or mean differences (MDs) with 95% confidence intervals.

**Results:**

Nine RCTs (2,420 women) were included, most with GDM and some with T2DM. Moderate-quality evidence indicated reduced stillbirth risk with M+I (6 RCTs, 2,196 participants; RR 0.36, 95% CI 0.14–0.90; NNT 111). Low-quality evidence suggested lower risks of gestational hypertension (4 RCTs; RR 0.68, 95% CI 0.48–0.97) and neonatal hypoglycemia (7 RCTs; RR 0.49, 95% CI 0.30–0.80). No significant differences were found for cesarean section, preterm delivery, or other neonatal outcomes. Heterogeneity, baseline imbalances, and small samples limited certainty.

**Conclusions:**

M+I may reduce stillbirth and some adverse outcomes compared with insulin alone, but most evidence remains low certainty. Further high-quality RCTs are needed.

**Registered in PROSPERO**: CRD42024617330

## Introduction

According to the World Health Organization, up to 10-12% of pregnancies are affected by diabetes. These numbers include patients with type 1 diabetes, type 2 diabetes (T2DM) related to obesity and insulin resistance, and gestational diabetes (GDM).^(1)^ GDM is the term used when the diagnosis of glucose intolerance is detected only during pregnancy and it is primarily related to increased levels of hormones that counteract insulin action, such as human placental lactogen and cortisol leading to insulin resistance. To compensate, β-cells increase insulin secretion, but failure to meet the heightened demand results in hyperglycemia.^(2-4)^

Recent evidence indicates that gestational diabetes is a heterogeneous condition resulting from varying degrees of insulin resistance and β-cell dysfunction, with some women showing a predominant defect in insulin secretion and an inadequate secretory response to the metabolic demands of pregnancy. This predominantly insulin-resistant profile provides a biological rationale for the use of metformin, which improves insulin sensitivity and complements insulin therapy when needed.^(5)^

Insulin is recommended worldwide as the standard approach to diabetes in pregnancy^(6,7)^ and is effective in reducing most diabetes-related complications.^(8-11)^ However, this treatment is associated with an increased incidence of hypertensive disorders and episodes of hypoglycemia in both mother and fetus.^(12-14)^ Despite adequate adherence to the recommended insulin therapy, one-third of pregnancies with diabetes still have complications related to poor glycemic control.^(15,16)^

Several studies have investigated the use of oral antidiabetic drugs, such as metformin, for use in pregnancies affected by diabetes.^(9,14,17,18)^ The use of metformin during pregnancy is considered safe, with few side effects, and preferred by the users, probably because it does not require injections.^(11,19,20)^ The mechanism of action is reducing insulin sensitivity and inhibiting hepatic glucose production.^(21-23)^ It is important to note that metformin crosses the placenta freely,^(10)^ and the short-term effects on these fetuses indicate that metformin is safe while the long-term effects are still unknown. Randomized studies have followed individuals exposed to metformin during pregnancy with conflicting and still inconclusive results regarding its safety,^(24-27)^ but in a recent metanalysis, no adverse effects were observed in children when comparing the long-term outcomes of this treatment with those of insulin.^(28)^

Metformin is increasingly being used in pregnancy despite uncertainties about its long-term safety. Guidelines recommendations are inconsistent; most recognize the benefits of metformin but do not yet recommend its use,^(29,30)^ which has led some authors to test the use of metformin in addition to insulin. The hypothesis is that this combination could provide adequate glycemic control with fewer adverse effects, allowing the use of lower doses of both drugs. Similarly, the Brazilian Diabetes Society (SBD) recognizes metformin as a therapeutic alternative for gestational diabetes when lifestyle measures are insufficient and insulin use is not feasible, and it also considers the combination of metformin with insulin in selected cases of gestational or type 2 diabetes. However, both international and national guidelines emphasize that evidence on the long-term safety of metformin exposure in utero remains limited.^(31-35)^

This systematic review aims to identify, assess, and summarize evidence on the effectiveness and safety of using metformin and insulin compared to insulin alone for pregnant women with T2DM or GDM, with stillbirth as the primary outcome.

## Methods

This systematic review with meta-analysis was conducted according to the Cochrane Collaboration guidelines for systematic reviews and meta-analyses. The results were reported as suggested by PRISMA (Preferred Reporting Items for Systematic Reviews and Meta-Analyses) guidelines.

### Search strategy

A search strategy was conducted in PubMed, Embase, and Cochrane Central to identify randomized trials involving pregnant women with diabetes. The protocols and findings of the included studies were also checked in the ClinicalTrials.gov registry. In PubMed, the following search was applied: (pregnant OR pregnancy OR gestation OR antenatal OR prenatal) AND (diabetes OR hyperglycemia OR "insulin resistance" OR GDM OR T2DM) AND (metformin OR glifage OR biguanide) AND (add OR adjunct OR plus) AND (random OR randomized OR randomised). A similar strategy was used in Embase and Cochrane Central. No date restrictions or filters were applied, and although English keywords were used, there were no formal language restrictions. The full search strings for all databases are provided in Supplementary Material - Table S1. The last search was performed in January 2025. No filters were applied, and no date or language restrictions were imposed.

### Inclusion and exclusion criteria

We considered eligible only the randomized clinical trials that included pregnant women with either type 2 diabetes (T2DM) or gestational diabetes (GDM), allocating part of the subjects to use insulin and metformin and part of the subjects only to insulin. We considered eligible only the randomized clinical trials that included pregnant women with either type 2 diabetes (T2DM) or gestational diabetes (GDM), allocating part of the subjects to use insulin and metformin and part of the subjects only to insulin. The primary outcome was stillbirth. Secondary outcomes included maternal outcomes (gestational hypertension, preeclampsia, maternal hypoglycemia); delivery outcomes (preterm delivery, cesarean section, gestational age at delivery); and neonatal outcomes (congenital malformations, birth weight, small for gestational age [SGA], large for gestational age [LGA], macrosomia, neonatal death, neonatal hypoglycemia, respiratory distress syndrome [RDS], transient tachypnea of the newborn [TTN], and neonatal intensive care unit [NICU] admission). We didn't consider eligible the trials that only included pregnant women with type 1 diabetes. Although there were no language restrictions in the literature search, we used English keywords. We didn't consider eligible the trials that only included pregnant women with type 1 diabetes.

### Study selection

Articles were screened in two stages (title/abstract and then full text) independently by two authors (CH and BVN), with disagreements resolved by discussion.

### Data extraction

Data were collected on maternal and fetal outcomes, including stillbirth (primary outcome); maternal outcomes (gestational hypertension, preeclampsia, maternal hypoglycemia); delivery outcomes (preterm delivery, cesarean section, gestational age at delivery); and neonatal outcomes (congenital malformations, birth weight, SGA, LGA, macrosomia, neonatal death, neonatal hypoglycemia, RDS, TTN, and NICU admission). Data regarding baseline characteristics of the samples included the type of diabetes, number of patients in each group, gestational age at enrollment, body mass index (BMI), and glycated hemoglobin levels. No assumptions were made; data were extracted directly as reported in the included studies. Two authors (CH and BVN) independently extracted and verified all data to ensure accuracy.

### Risk assessment

Risk of bias was assessed independently by two authors (CH and BVN) using the Cochrane RoB2 tool. Differences were resolved by discussion with a third author (WPM).

### Statistical analysis

Cochrane RevMan 5.4 software was used for statistical analysis. Dichotomous outcomes were expressed as relative risk (RR) with a 95% confidence interval, whereas continuous outcomes were expressed as mean difference (MD) with a 95% confidence interval. Heterogeneity was assessed using Cochran's Q and I² tests, using a random-effects model. "Summary of Findings" (SOF), was constructed with the objective of providing a transparent and readily comprehensible representation of the results, emphasizing the most significant findings and their clinical implications. The number needed to treat (NNT) was calculated as the inverse of the absolute risk reduction (ARR). ARR was obtained by subtracting the risk in the metformin + insulin group from the risk in the insulin-only group. This measure was included to complement relative estimates (RR) by providing absolute effect sizes.

### Subgroup analyses

Subgroup analyses were conducted to identify potential sources of heterogeneity and to assess the robustness of the overall results. The subgroup analyses were based on two factors: the timing of the introduction of insulin into treatment and the type of diabetes. Specifically, we compared cases in which insulin was initiated at randomization (also referred as at diagnosis) with cases in which insulin was introduced only when glycemic control was inadequate with metformin alone. In addition, a comparison was made between T2DM and GDM. To assess the robustness of the pooled estimates, two sensitivity analyses were performed. The first excluded studies classified as having a high risk of bias, and the second excluded both high-risk and *some concerns* studies.

### Quality of the evidence and the strength of the recommendations

We used GRADEpro GDT (Guideline Development Tool) software, which applies the GRADE (Grading of Recommendations Assessment, Development and Evaluation) system, to assess the quality of evidence and the strength of recommendations. Relevant data were extracted from included studies and entered GRADEpro, which assessed results for bias, inconsistency, imprecision, indirectness, and publication bias. Evidence quality was graded into four levels (high, moderate, low, and very low).

### Reporting bias assessment

To assess potential publication bias, we reviewed the information available for the included trials and contacted study authors to request additional or unpublished data, when necessary. Funnel plots were used to explore possible asymmetries; however, the small number of included studies precluded a reliable assessment of publication bias.

### Registration

This systematic review was registered in PROSPERO under the identification number CRD42024617330 on December 2, 2024.

## Results

### Study selection

**Figure 1:**
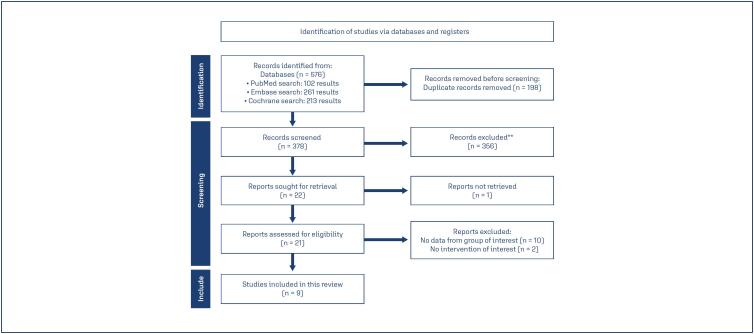
PRISMA flow diagram of study process screening and selection

The initial search yielded a total of 576 records, with 378 remaining for screening after removing duplicates. There were 356 records excluded on different grounds, leaving 22 records to be retrieved. One of these reports was unavailable. Of the remaining 21 reports assessed for eligibility, 12 were excluded because they did not present data on the group of interest or outcomes of interest (Supplementary Material Table 2S). This left 9 studies for inclusion. The selection process is detailed in figure 1. A detailed overview of the study populations are presented in chart 1.

**Chart 1:** Characteristics of the included studies and participants

Study	Diabetes type	Participants		Inclusion (wk)		BMI		HbA1c	
		I+M	Insulin	I+M	Insulin	I+M	Insulin	I+M	Insulin
Studies starting both metformin and Insulin at randomization vs. Insulin only									
Boggess et al. (MOMPOD) (2023)^(36)^	T2DM	397	397	< 23		36.4±8.0	36.3±8.9	7.7±2.0	7.7±2
Ibrahim et al. (2014)*^(37)^	GDM/T2DM	46	44	28.7±3.71		31.83±3.23		NI	NI
Feig et al. (2020)^(38)^ (MiTy)	T2DM	253	249	16.5±4.0	16.4±3.8	35.0 ± 7.1	35.0 ± 7.1	6.4±1.2	6.4±1.1
Studies starting metformin at randomization, adding insulin if poor glycemic control vs. Insulin only									
Ainuddin et al. (2015)^(34)^	T2DM	90	100	10.1±4.8	9.6±5.2	33.6±3.9	32.9±4.0	NI	NI
Ainuddin et al. (2015)^(35)^	GDM	32	75	29.7±1,6	29.2±1,5	NI	NI	5.3±0.5	5.2±0.6
Ashoush et al. (2016)^(39)^	GDM	11	48	NI	27.8±1.4	32.5±1.0	31.4±1.5	6.0±0.5	5.8±0.6
Dasari et al. (2023)^(40)^	GDM	13	80	NI	NI	30.9	29.2±14	NI	NI
Ijäs et al. (2011)^(41)^	GDM	15	32	30±4.9	30±4.0	31.5±6.5	30.8±5.4	5.9±0.4	5.9±0.4
Rowan et al. (2008)^(19)^ (MiG)	GDM	168	370	+ 20 days	30.1±3.2	36.7±9.3	34.6±7.2	5.9±0.7	5.9±0.4

BMI = Body Mass Index (kg/m²); GMD = Gestational Diabetes Mellitus; HbA1c = Glycated Hemoglobin (%); T2DM = Type 2 Diabetes I+M = Insulin + Metformin; I only = Insulin only; NA = not available;

* Trial conducted with patients who failed insulin at a dose of 1.12U/kg

### Risk of bias assessments

**Figure 2:**
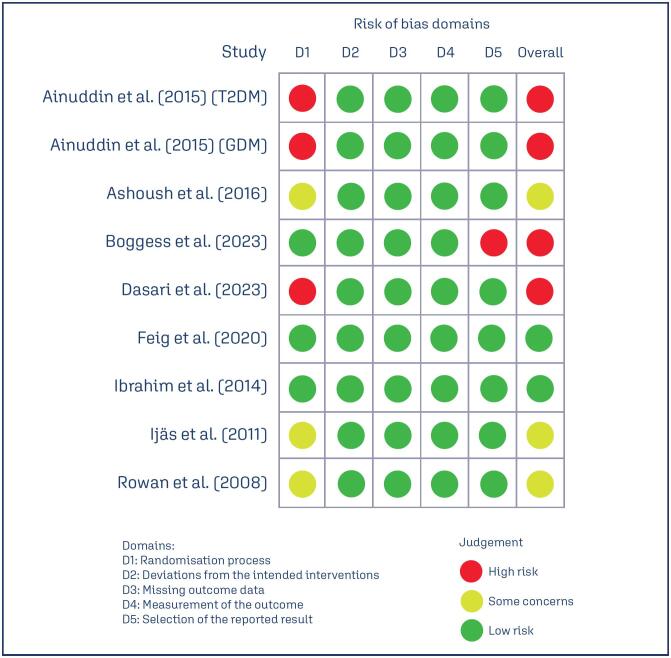
Risk of bias assessment

The present review included nine studies. Two studies were deemed to have a low risk of bias across all domains, while three studies were identified as having a high risk of bias with respect to the randomization process and one study was identified as having a high risk of bias with respect to the selection of reported results. The overall risk was therefore considered to be high or moderate. The summary of the risk of bias assessment domains is presented in figure 2.

### Main comparison

**Figure 3:**
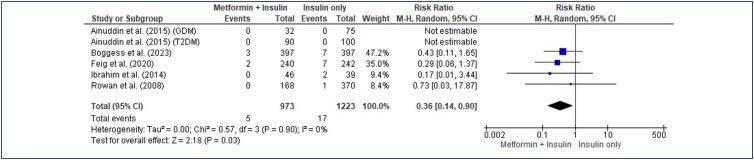
Forest plot of comparison: Insulin+metformin x Insulin only, outcome: Stillbirth

We found that metformin combined with insulin significantly reduces the risk of stillbirth compared with insulin alone (RR 0.36, 95% CI 0.14–0.90; I² = 0%; NNT = 111), with moderate-quality evidence. Among maternal outcomes, low-quality evidence suggested a lower risk of gestational hypertension (RR 0.68, 95% CI 0.48–0.97; I²=0%; NNT = 34), while no significant differences were observed for preeclampsia or maternal hypoglycemia. For delivery outcomes, no significant differences were found for preterm delivery, cesarean section, or gestational age at delivery. Regarding neonatal outcomes, low-quality evidence indicated a reduced risk of neonatal hypoglycemia (RR 0.49, 95% CI 0.30–0.80; I²=62%; NNT = 11). No significant differences were found for congenital malformations, birth weight, SGA, LGA, macrosomia, neonatal death, or respiratory complications (RDS and TTN) and NICU admission. The summarized estimates of the main comparison (Metformin + Insulin vs. Insulin) are presented in chart 2, and the forest-plots for the main outcome is presented in figure 3.

**Chart 2:** Pooled estimated observed for the main outcomes in the comparison Metformin + Insulin vs. Insulin only

Outcome	Participants	Relative effect	Absolute effects (95% CI)			NNT	Certainty
	(studies)	(95% CI)	Insulin	M+I	Difference		
Hypertension	1573	RR 0.68	90 per 1,000	61 per 1,000	29 fewer per 1,000	34	⨁⨁◯◯
	(4 RCTs)	(0.48 to 0.97)		(43 to 87)	(from 47 fewer to 3 fewer)		Low ^a^
Preeclampsia	1573	RR 1.07	104 per 1,000	112 per 1,000	7 more per 1,000	143	⨁◯◯◯
	(4 RCTs)	(0.71 to 1.60)		(74 to 167)	(from 30 fewer to 63 more)		Very low ^f^
Severe Maternal Hypoglicemia	1264	RR 1.03	13 per 1,000	13 per 1,000	0 fewer per 1,000		⨁◯◯◯
	(2 RCTs)	(0.39 to 2.76)		(5 to 35)	(from 8 fewer to 22 more)		Very low ^f^
Preterm Delivery	1814	RR 1.25	96 per 1,000	120 per 1,000	24 more per 1,000	42	⨁⨁◯◯
	(3 RCTs)	(0.95 to 1.64)		(91 to 158)	(from 5 fewer to 62 more)		Low ^b^
Caesarean Section	1695	RR 0.88	630 per 1,000	555 per 1,000	76 fewer per 1,000	13	⨁◯◯◯
	(6 RCTs)	(0.74 to 1.04)		(466 to 655)	(from 164 fewer to 25 more)		Very low ^f^
Gestational Age at Delivery	1612				-0.07 weeks		⨁◯◯◯
	(5 RCTs)				(-0.31 to 0.17 weeks)		Very low ^d^
Stillbirth	2196	RR 0.36	14 per 1,000	5 per 1,000	9 fewer per 1,000	111	⨁⨁⨁◯
	(6 RCTs)	(0.14 to 0.90)		(2 to 13)	(from 12 fewer to 1 fewer)		Moderate ^a^
Congenital Malformations	1282	RR 0.94	35 per 1,000	33 per 1,000	2 fewer per 1,000	500	⨁◯◯◯
	(3 RCTs)	(0.42 to 2.09)		(15 to 72)	(from 20 fewer to 38 more)		Very low ^f^
Birthweight	1672				-0.1 kg		⨁◯◯◯
	(6 RCTs)				(-0.25 to 0.04 kg)		Very low ^f^
Small for Gestational Age	1559	RR 1.94	60 per 1,000	116 per 1,000	56 more per 1,000	18	⨁⨁◯◯
	(4 RCTs)	(1.07 to 3.51)		(64 to 211)	(from 4 more to 151 more)		Low ^c^
Large for Gestational Age	1638	RR 0.82	344 per 1,000	282 per 1,000	62 fewer per 1,000	16	⨁◯◯◯
	(5 RCTs)	(0.65 to 1.04)		(223 to 358)	(from 120 fewer to 14 more)		Very low ^f^
Macrossomia	629	RR 0.72	202 per 1,000	146 per 1,000	57 fewer per 1,000	18	⨁⨁◯◯
	(3 RCTs)	(0.51 to 1.03)		(103 to 208)	(from 99 fewer to 6 more)		Low ^b^
Neonatal Death	1364	RR 1.72	4 per 1,000	7 per 1,000	3 more per 1,000	333	⨁◯◯◯
	(3 RCTs)	(0.17 to 17.16)		(1 to 73)	(from 4 fewer to 68 more)		Very low^f^
Neonatal Hypoglycemia	2263	RR 0.49	171 per 1,000	84 per 1,000	87 fewer per 1,000	11	⨁⨁◯◯
	(7 RCTs)	(0.30 to 0.80)		(51 to 137)	(from 119 fewer to 34 fewer)		Low ^c^
Respiratory Distress Syndrome	1584	RR 0.71	54 per 1,000	38 per 1,000	16 fewer per 1,000	63	⨁⨁◯◯
	(5 RCTs)	(0.44 to 1.15)		(24 to 62)	(from 30 fewer to 8 more)		Low ^b^
Transient Tachypnea of Newborn	835	RR 0.99	50 per 1,000	49 per 1,000	0 fewer per 1,000		⨁◯◯◯
	(3 RCTs)	(0.27 to 3.61)		(13 to 179)	(from 36 fewer to 129 more)		Very low ^e^
NICU admission	1694	RR 0.72	377 per 1,000	271 per 1,000	105 fewer per 1,000	10	⨁◯◯◯
	(6 RCTs)	(0.47 to 1.10)		(177 to 414)	(from 200 fewer to 38 more)		Very low ^d^

Notes: 95% CI = 95% confidence interval; RR = Risk Ratio determined by using the Mantel-Haenszel method and a random-effects model; NICU = neonatal intensive care unit; NNT = Number Needed to Treat Evaluation of que certainty level: a = downgraded one level by serious imprecision; b = downgraded two levels by very serious imprecision; c = downgraded one level because of inconsistency and one level because of serious imprecision; d = downgraded one level because of serious risk of bias, one level because of serious inconsistency and two levels because of very serious imprecision; e = downgraded one level because of serious risk of bias, one level because of serious inconsistency and one level because of serious imprecision; f = downgraded one level because of serious inconsistency and two levels because of very serious imprecision.

### Subgroup analysis

The main results for the subgroups analysis separating the studies by the time of insulin addition (Insulin added by the time of randomization vs. Insulin added only when glycemic control was not satisfactory with metformin only) are presented in Supplementary Material Table 3S.

The main results for the subgroups analysis separating the studies by type of DM (GDM vs. T2DM) are presented in Supplementary Material Table 4S. The subgroup analyses were not able to explain the observed heterogeneity observed for some outcomes. For the primary outcome (stillbirth), the certainty of evidence remained moderate across subgroups. In the subgroup where women received metformin plus insulin at randomization, neonatal outcomes included lower birthweight (MD –0.14 kg, 95% CI –0.24 to –0.05) and a reduced risk of large-for-gestational-age infants (RR 0.72, 95% CI 0.61–0.85) compared with insulin alone. In the subgroup analysis by diabetes type, among women with T2DM, newborns had lower birthweight (MD –0.16 kg, 95% CI –0.24 to –0.08) and a reduced risk of transient tachypnea of the newborn (RR 0.37, 95% CI 0.15–0.89).

### Sensitivity analysis

Sensitivity analyses excluding studies at high risk of bias, and subsequently those at high risk or with some concerns, yielded pooled estimates consistent in magnitude and direction with the main analyses. No significant differences were observed.

## Discussion

In the overall population analyzed, we observed moderate quality evidence that the combining Metformin and Insulin might reduce the risk of stillbirth (RR 0.36, 95% CI 0.14–0.90; I²=0%; NNT 111), when compared to Insulin alone. For the other outcomes, the evidence was deemed as being low or very low.

Among maternal outcomes, our data showed a significant reduction in gestational hypertension, with low quality of evidence (RR 0.68, 95% CI 0.48–0.97; I²=0%; NNT = 34). Previous research has indicated that metformin alone is an effective method of reducing preeclampsia^(8)^ and maternal hypertension.^(32)^ However our findings did not corroborate this observation.

Regarding delivery outcomes, no differences were observed in cesarean section rates,^(8,13,33)^ and there was no consistent evidence of an effect on preterm birth or gestational age at delivery.

For neonatal outcomes, our data shows a significant reduction in neonatal hypoglycemia (RR 0.49, 95% CI 0.30–0.80; I²=62%; NNT = 11) with low quality of evidence. Fetal hypoglycemia was evaluated with the use of metformin alone,^(8,13,32,33)^ with no differences from our findings. We observed a non-significant trend towards reduced NICU admissions and reduced fetal weight, but this was more pronounced with metformin alone.^(8,13)^

Although the relative reduction in stillbirth was substantial, the absolute risk difference was small because stillbirth is a rare event, resulting in a high number needed to treat (NNT). In contrast, maternal hypertension and neonatal hypoglycemia are more frequent outcomes, which therefore yield lower NNT values.

Subgroup analyses separated patients by timing of insulin initiation and by type of diabetes. While these analyses indicated potential benefits, the sources of heterogeneity remain unclear. Consequently, the clinical application of these findings should be interpreted with caution. Further research is required to elucidate the sources of the observed heterogeneity.

The quality of the studies included in our analysis raises important concerns. Ainuddin et al. (T2DM)^(34)^ and Ainuddin et al. (GDM)^(35)^ were identified as having a high risk of bias due to a lack of allocation concealment. Several concerning differences were identified between the populations of Ashoush et al.^(39)^ and Rowan et al.^(19)^ in terms of BMI and HbA1c. Furthermore, differences in BMI were also evident in the Dasari et al.^(40)^ and Ijäs et al.^(41)^ studies. In the studies by Ainuddin et al. (T2DM),^(34)^ Ainuddin et al. (GDM),^(35)^ Ashoush et al.,^(39)^ Dasari et al.,^(40)^ Ijäs et al.,^(41)^ and Rowan et al.,^(19)^ the metformin + insulin group was formed after the initial randomization to insulin or metformin. Patients who failed monotherapy were switched to the metformin + insulin group, potentially selecting more severe cases and resulting in different numbers of patients in each group (625 for insulin and 316 for metformin + insulin).

In the Boggess et al.,^(36)^ Ibrahim et al.^(37)^ and Feig et al.^(38)^ trials, participants were randomized to investigate the comparative efficacy of insulin alone versus insulin plus metformin. The Ibrahim trial selected patients who exhibited inadequate glycemic control despite an insulin dose of 1.12 U/kg. The patients had been diagnosed with type 2 diabetes for a median of four years (range, 1–15), resulting in the selection of more severe cases.

The studies by Ainuddin et al.,^(34)^ Boggess et al.,^(36)^ and Feig et al.^(38)^ included patients with T2DM but did not provide information on the duration of diabetes, the type of treatment received prior to pregnancy, or the presence of comorbidities. As these factors are known to exert a significant influence on gestational outcomes, their absence limits the interpretability of the findings.

Boggess's et al.^(36)^ trial shows important internal inconsistencies that undermine the reliability of its findings. Several maternal and neonatal outcomes were reported with discrepant values between the main text, the supplementary appendix, and the trial registry, with no clear explanation for the differences. Definitions of key outcomes, such as hypertensive disorders, were also not applied consistently across sources. Furthermore, the flowchart presents totals that do not match the reported results.

A notable strength of our systematic review and meta-analysis is that it enabled the aggregation of studies reporting stillbirth, revealing a significant risk reduction (RR 0.36, 95% CI 0.14–0.90; I² = 0%; NNT = 111) for the combined metformin plus insulin therapy. While individual randomized studies did not reach statistical significance, our pooled analysis provided sufficient power to detect a consistent reduction in stillbirth risk.^(19,25,32,34-37)^

Previous meta-analyses did not fully explore this outcome in this context. A meta-analysis published in 2021 addresses the efficacy and safety of metformin alone or added to insulin. However, this study identified only four studies that used metformin added to insulin out of 21 articles included. Therefore, this meta-analysis is more concerned with metformin than with the practice of adding metformin to insulin to treat diabetes in pregnancy.^(9)^

However, some included trials presented methodological limitations, such as high or unclear risk of bias, small sample sizes, and baseline imbalances, which reduce the overall certainty of evidence and should be considered when interpreting these findings.

## Conclusion

The combination of metformin and insulin has been found to confer beneficial outcomes for both mothers and infants and represents a safe option for pregnant women with diabetes. The effects of this treatment appear to vary according to the timing of its initiation and the type of diabetes being treated. Nonetheless, the certainty of evidence remains limited due to imprecision and methodological weaknesses in some of the included trials. Therefore, while the overall findings are positive, they should be applied with caution until further high-quality randomized studies confirm these effects.

## Data Availability

The research data are described in the article presented.
