# Supplementary material for: Metformin + Insulin vs. Insulin for GDM and T2DM during pregnancy: systematic review and meta-analysis
Source: Rev Bras Ginecol Obstet. 2026 May 12;48:e-rbgo18. doi: 10.61622/rbgo/2026rbgo18 (PMC13399377; doi:10.61622/rbgo/2026rbgo18)
Supplement: Supplementary Material [file 1806-9339-rbgo-48-e-rbgo18-Supp01.pdf]

Supplementary Material

Table S1. Search strategies

**PubMed**
(pregnant OR pregnancy OR gestation OR antenatal OR prenatal) AND ("insulin-resistant" OR diabetes OR hyperglycemia OR "insulin resistance" OR GDM OR T2DM) AND (metformin OR glifage OR biguanide) AND (add OR "first line" OR adding OR adjunct OR supplement OR supplemental OR supplemented OR plus) AND (random OR randomized OR randomised)
**Embase**
(pregnant OR 'pregnancy'/exp OR pregnancy OR 'gestation'/exp OR gestation OR antenatal OR 'prenatal'/exp OR prenatal) AND ('insulin-resistant' OR 'diabetes'/exp OR diabetes OR 'hyperglycemia'/exp OR hyperglycemia OR 'insulin resistance'/exp OR 'insulin resistance' OR gdm OR 't2dm'/exp OR t2dm) AND ('metformin'/exp OR metformin OR 'glifage'/exp OR glifage OR 'biguanide'/exp OR biguanide) AND (add OR 'first line' OR adding OR adjunct OR supplement OR supplemental OR supplemented OR 'plus'/exp OR plus) AND (random OR randomized OR randomised)
**Cochrane Central**
(pregnant OR pregnancy OR gestation OR antenatal OR prenatal) AND ("insulin-resistant" OR diabetes OR hyperglycemia OR "insulin resistance" OR GDM OR T2DM) AND (metformin OR glifage OR biguanide) AND (add OR "first line" OR adding OR adjunct OR supplement OR supplemental OR supplemented OR plus) AND (random OR randomized OR randomised)

Table 2S. Excluded studies

Study	Exclusion reason
Ainuddin (2012)[1]	No results could be retrieved from the interest group/poster-abstract
Anzolin et al. (2018)[2]	Poster-abstract
Ardilouze et al. (2014)[3]	Poster-abstract
Beyuo et al. (2015)[4]	No results could be retrieved from the interest group
Dunne et al. (2023)[5]	Ongoing study
Hickman et al. (2013)[6]	No results could be retrieved from the interest group
Muhsen et al. (2022)[7]	No results could be retrieved from the interest group
Niromanesh et al. (2012)[8]	No results could be retrieved from the interest group
Rademaker et al. (2024)[9]	Study protocol
Roy et al. (2018)[10]	No results could be retrieved from the interest group
Spaulonci et al. (2013)[11]	No results could be retrieved from the interest group
Reynolds et al. (2017)[12]	Feasibility pilot study

Table 3S. Subgroup analysis by timming to iniciate metformin

Outcome or Subgroup	Studies	N	Statistical Method	Effect Estimate
Hypertension	4	1573	Risk Ratio (M-H, Random, 95% CI)	0.68 [0.48, 0.97]
Add at fail	2	297	Risk Ratio (M-H, Random, 95% CI)	0.60 [0.40, 0.92]
Add at randomization	2	1276	Risk Ratio (M-H, Random, 95% CI)	0.90 [0.47, 1.70]
Preeclampsia	4	1573	Risk Ratio (M-H, Random, 95% CI)	1.07 [0.71, 1.60]
Add at fail	2	297	Risk Ratio (M-H, Random, 95% CI)	0.56 [0.28, 1.14]
Add at randomization	2	1276	Risk Ratio (M-H, Random, 95% CI)	1.31 [0.96, 1.79]
Caesarean Section	6	1695	Risk Ratio (M-H, Random, 95% CI)	0.88 [0.74, 1.04]
Add at fail	3	390	Risk Ratio (M-H, Random, 95% CI)	0.78 [0.51, 1.19]
Add at randomization	3	1305	Risk Ratio (M-H, Random, 95% CI)	0.95 [0.84, 1.06]
Gestational Age at Delivery	5	1612	Std. Mean Difference (IV, Random, 95% CI)	-0.05 [-0.24, 0.14]
Add at fail	2	297	Std. Mean Difference (IV, Random, 95% CI)	-0.33 [-0.72, 0.07]
Add at randomization	3	1315	Std. Mean Difference (IV, Random, 95% CI)	0.05 [-0.10, 0.20]
Birthweight	6	1672	Std. Mean Difference (IV, Random, 95% CI)	-0.20 [-0.41, 0.02]
Add at fail	3	362	Std. Mean Difference (IV, Random, 95% CI)	-0.12 [-0.79, 0.56]
Add at randomization	3	1310	Std. Mean Difference (IV, Random, 95% CI)	-0.23 [-0.34, -0.12]
Small for Gestational Age	4	1559	Risk Ratio (M-H, Random, 95% CI)	1.94 [1.07, 3.51]
Add at fail	2	297	Risk Ratio (M-H, Random, 95% CI)	3.79 [1.22, 11.75]
Add at randomization	2	1262	Risk Ratio (M-H, Random, 95% CI)	1.44 [0.89, 2.35]
Large for Gestational Age	5	1638	Risk Ratio (M-H, Random, 95% CI)	0.82 [0.65, 1.04]
Add at fail	3	362	Risk Ratio (M-H, Random, 95% CI)	1.09 [0.72, 1.65]
Add at randomization	2	1276	Risk Ratio (M-H, Random, 95% CI)	0.72 [0.61, 0.85]
Neonatal Hypoglycemia	7	2263	Risk Ratio (M-H, Random, 95% CI)	0.49 [0.30, 0.80]
Add at fail	4	928	Risk Ratio (M-H, Random, 95% CI)	0.43 [0.18, 1.00]
Add at randomization	3	1335	Risk Ratio (M-H, Random, 95% CI)	0.51 [0.24, 1.09]
NICU	6	1694	Risk Ratio (M-H, Random, 95% CI)	0.72 [0.47, 1.10]
Add at fail	4	489	Risk Ratio (M-H, Random, 95% CI)	0.56 [0.32, 0.98]
Add at randomization	2	1205	Risk Ratio (M-H, Random, 95% CI)	0.94 [0.81, 1.09]

Table 4S. Subgroup analysis by diabetes type

Outcome or Subgroup	Studies	N	Statistical Method	Effect Estimate
Caesarean Section	6	1695	Risk Ratio (M-H, Random, 95% CI)	0.88 [0.74, 1.04]
GDM	3	282	Risk Ratio (M-H, Random, 95% CI)	1.01 [0.81, 1.26]
T2DM	3	1413	Risk Ratio (M-H, Random, 95% CI)	0.83 [0.65, 1.05]
Gestational Age at Delivery	5	1612	Std. Mean Difference (IV, Random, 95% CI)	-0.05 [-0.24, 0.14]
GDM	2	194	Std. Mean Difference (IV, Random, 95% CI)	-0.10 [-1.00, 0.80]
T2DM	3	1418	Std. Mean Difference (IV, Random, 95% CI)	-0.01 [-0.11, 0.10]
Birthweight	6	1672	Std. Mean Difference (IV, Random, 95% CI)	-0.20 [-0.41, 0.02]
GDM	3	254	Std. Mean Difference (IV, Random, 95% CI)	-0.10 [-0.87, 0.67]
T2DM	3	1418	Std. Mean Difference (IV, Random, 95% CI)	-0.22 [-0.33, -0.12]
Small for Gestational Age	4	1559	Risk Ratio (M-H, Random, 95% CI)	1.94 [1.07, 3.51]
GDM	1	107	Risk Ratio (M-H, Random, 95% CI)	2.34 [0.73, 7.54]
T2DM	3	1452	Risk Ratio (M-H, Random, 95% CI)	1.94 [0.92, 4.08]
Large for Gestational Age	5	1638	Risk Ratio (M-H, Random, 95% CI)	0.82 [0.65, 1.04]
GDM	2	172	Risk Ratio (M-H, Random, 95% CI)	1.03 [0.42, 2.51]
T2DM	3	1466	Risk Ratio (M-H, Random, 95% CI)	0.81 [0.62, 1.06]
Neonatal Hypoglycemia	7	2263	Risk Ratio (M-H, Random, 95% CI)	0.49 [0.30, 0.80]
GDM	4	820	Risk Ratio (M-H, Random, 95% CI)	0.39 [0.15, 1.02]
T2DM	3	1443	Risk Ratio (M-H, Random, 95% CI)	0.52 [0.26, 1.03]
Transient Tachypnea of Newborn	3	835	Risk Ratio (M-H, Random, 95% CI)	0.99 [0.27, 3.61]
GDM	2	645	Risk Ratio (M-H, Random, 95% CI)	1.71 [0.63, 4.65]
T2DM	1	190	Risk Ratio (M-H, Random, 95% CI)	0.37 [0.15, 0.89]
NICU	6	1694	Risk Ratio (M-H, Random, 95% CI)	0.72 [0.47, 1.10]
GDM	3	299	Risk Ratio (M-H, Random, 95% CI)	0.71 [0.41, 1.22]
T2DM	3	1395	Risk Ratio (M-H, Random, 95% CI)	0.71 [0.39, 1.27]

References

1. Ainuddin J. Metformin: a safe alternative to insulin therapy in gestational diabetes. Int J Gynecol Obstet. 2012;119(S3):S270. doi: 10.1016/S0020-7292(12)60457-3
2. Anzolin G, Silva J, Wolff LC, Salles W, Souza M, Palmieri A. Use of metformin prophylatic in gestacional diabetes mellitus. Int J Gynecol Obstet. 2018;143 Suppl 3:718-9. doi: 10.1002/ijgo.12583
3. Ardilouze JL, Ménard J, Hivert MF, Houde G, Perron P, Moutquin JM, et al. Gestational diabetes mellitus: a randomized study comparing insulin therapy to a combination of half-maximal dosages of metformin and glyburide. Can J Diabetes. 2014;38(5):S23. doi: 10.1016/j.jcjd.2014.07.064
4. Beyuo T, Obed SA, Adjepong-Yamoah KK, Bugyei KA, Oppong SA, Marfoh K. Metformin versus insulin in the management of pre-gestational diabetes mellitus in pregnancy and gestational diabetes mellitus at the Korle Bu Teaching Hospital: a randomized clinical trial. PLoS One. 2015;10(5):e0125712. doi: 10.1371/journal.pone.0125712
5. Dunne FP, Alvarez-Iglesias A, Newman C, Smyth A, Browne M, Devane D, et al. A randomized placebo-controlled trial of the effectiveness of early metformin in addition to usual care in the reduction of gestational diabetes mellitus effects (EMERGE). Diabetes. 2023;72 Suppl 1:183-LB. doi: 10.2337/db23-183-LB
6. Hickman MA, McBride R, Boggess KA, Strauss R. Metformin compared with insulin in the treatment of pregnant women with overt diabetes: a randomized controlled trial. Am J Perinatol. 2013;30(6):483-90. doi: 10.1055/s-0032-1326994
7. Muhsen N, Al-Rubai S, Qahtan H. Metformin versus insulin in then managment of gestational diabetes mellitus. Indian J Forensic Med Toxicol. 2022;16(3):275-9. doi: 10.37506/ijfmt.v16i3.18297
8. Niromanesh S, Alavi A, Sharbaf FR, Amjadi N, Moosavi S, Akbari S. Metformin compared with insulin in the management of gestational diabetes mellitus: a randomized clinical trial. Diabetes Res Clin Pract. 2012;98(3):422-9. doi: 10.1016/j.diabres.2012.09.031
9. Rademaker D, de Wit L, Duijnhoven RG, Voormolen-van Münster DN, van der Post J, Bosmans JE, et al. Oral antihyperglycemic agent strategy versus insulin for gestational diabetes mellitus – a randomized controlled trial. Am J Obstet Gynecol. 2024;230(1):S23. doi: 10.1016/j.ajog.2023.11.045
10. Roy SB, Roy SB, Alam H, Chowdhury S, Saha S. The use of metformin versus insulin in the management of diabetes mellitus in pregnancy . a randomized control trial. J Indian Med Assoc. 2018;116(6):18-20.
11. Spaulonci CP, Bernardes LS, Trindade TC, Zugaib M, Francisco RP. Randomized trial of metformin vs insulin in the management of gestational diabetes. Am J Obstet Gynecol. 2013;209(1):34.e1-7. doi: 10.1016/j.ajog.2013.03.022
12. Reynolds RM, Denison FC, Juszczak E, Bell JL, Penneycard J, Strachan MW, et al. Glibenclamide and metfoRmin versus stAndard care in gEstational diabeteS (GRACES): a feasibility open label randomised trial. BMC Pregnancy Childbirth. 2017;17(1):316. doi: 10.1186/s12884-017-1505-3
